# Supplementary material for: Mitogenomic Analysis and Phylogenetic Implications for the Deltocephaline Tribe Chiasmini (Hemiptera: Cicadellidae: Deltocephalinae)
Source: Insects. 2024 Apr 8;15(4):253. doi: 10.3390/insects15040253 (PMC11050438; doi:10.3390/insects15040253)
Supplement: Supplementary file 1 [file insects-15-00253-s001.zip › insects-2878867-supplementary figures.pdf]

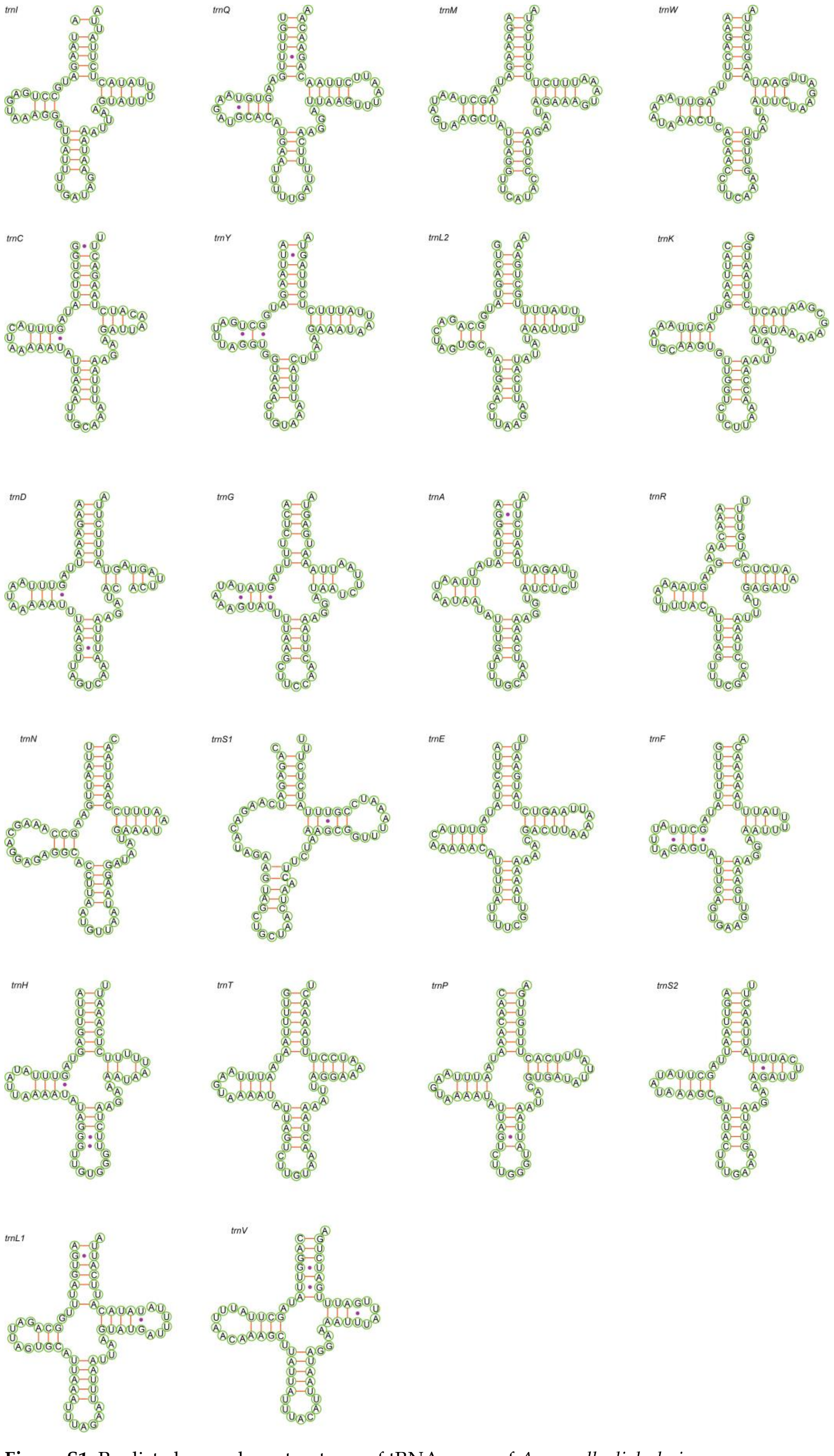

Figure S1. Predicted secondary structures of tRNA genes of *Aconurella diplachnis*.

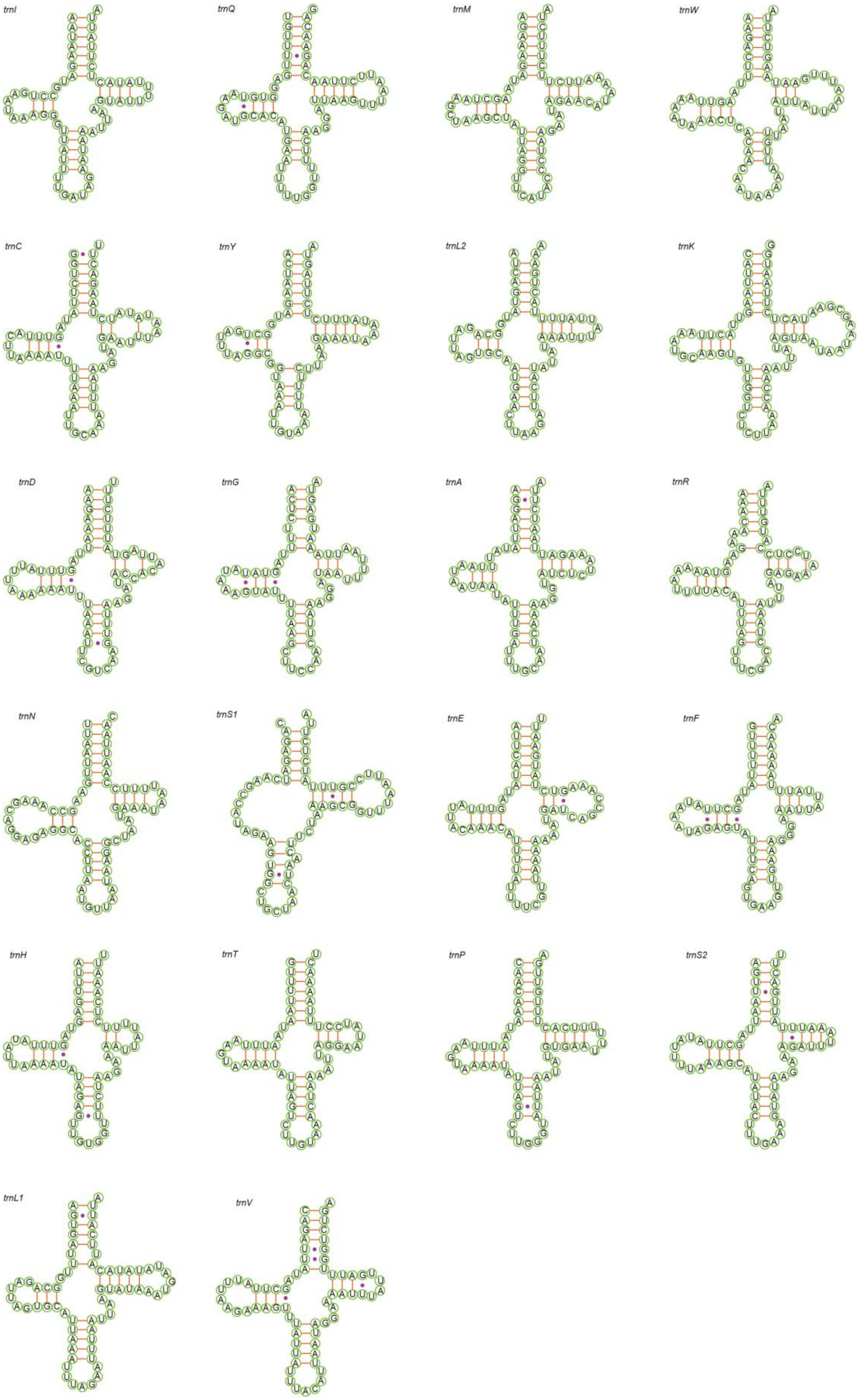

Figure S2. Predicted secondary structures of tRNA genes of *Aconurella montana*.

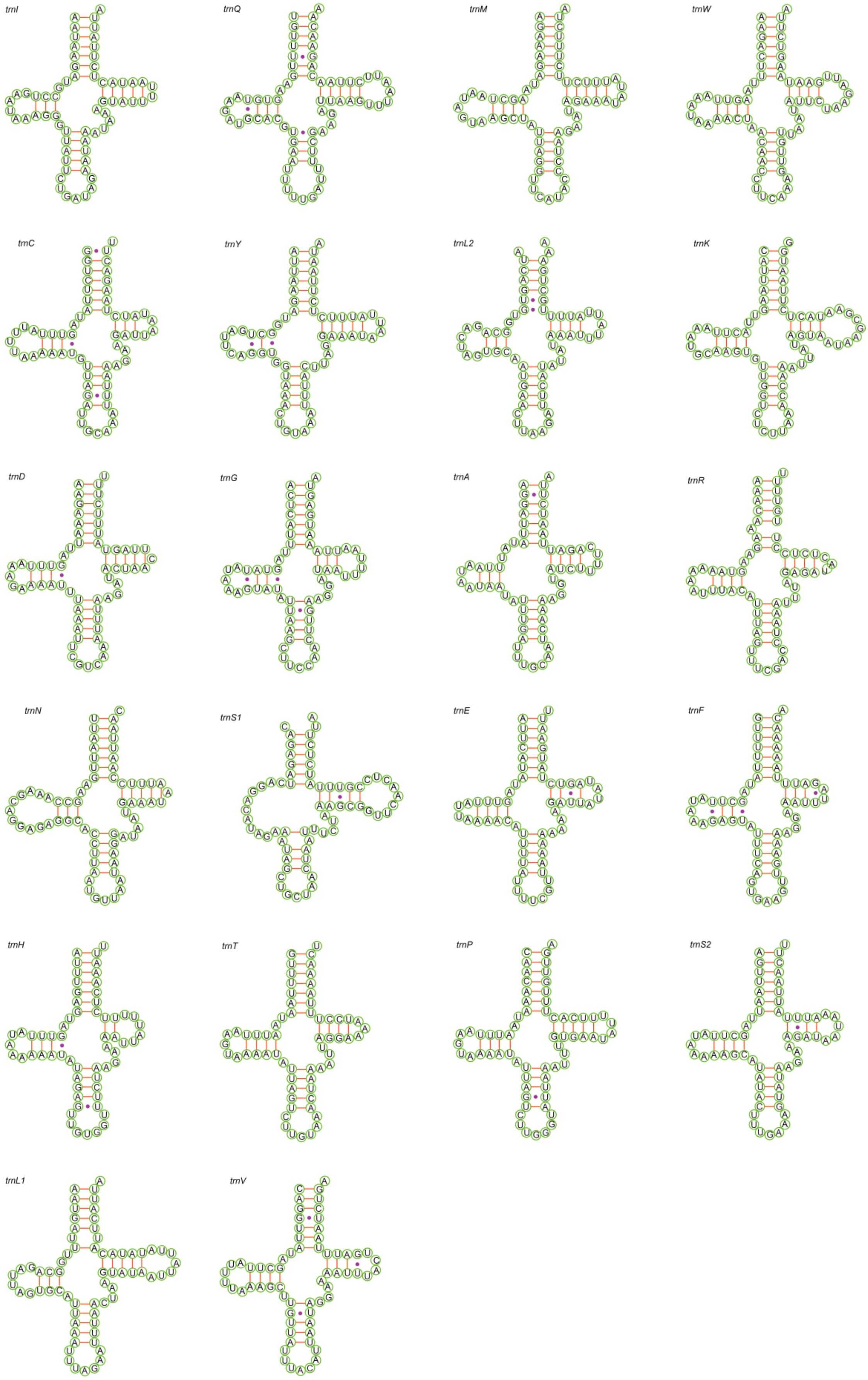

Figure S3. Predicted secondary structures of tRNA genes of *Aconurella prolixa*.

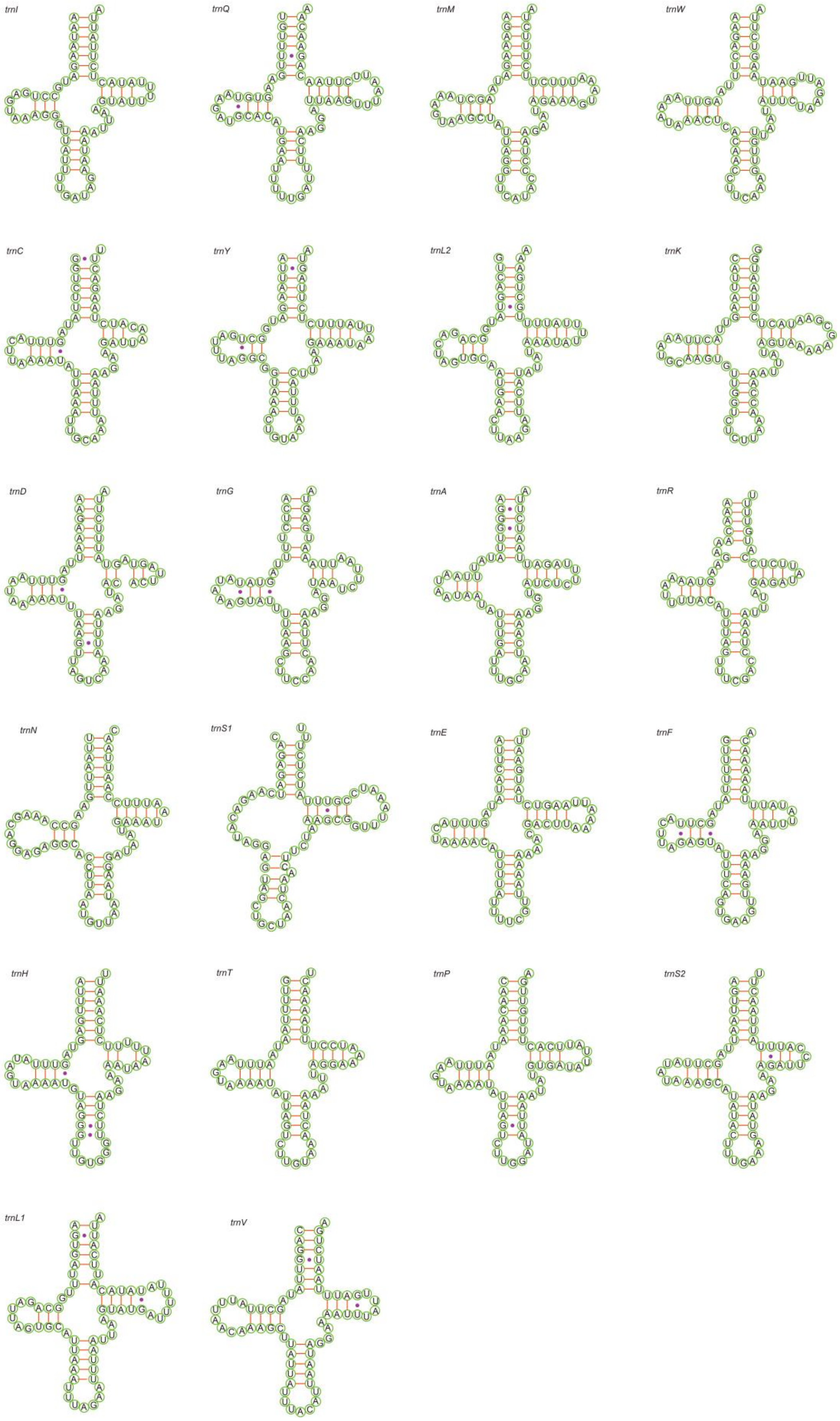

Figure S4. Predicted secondary structures of tRNA genes of *Aconurella sibirica*.

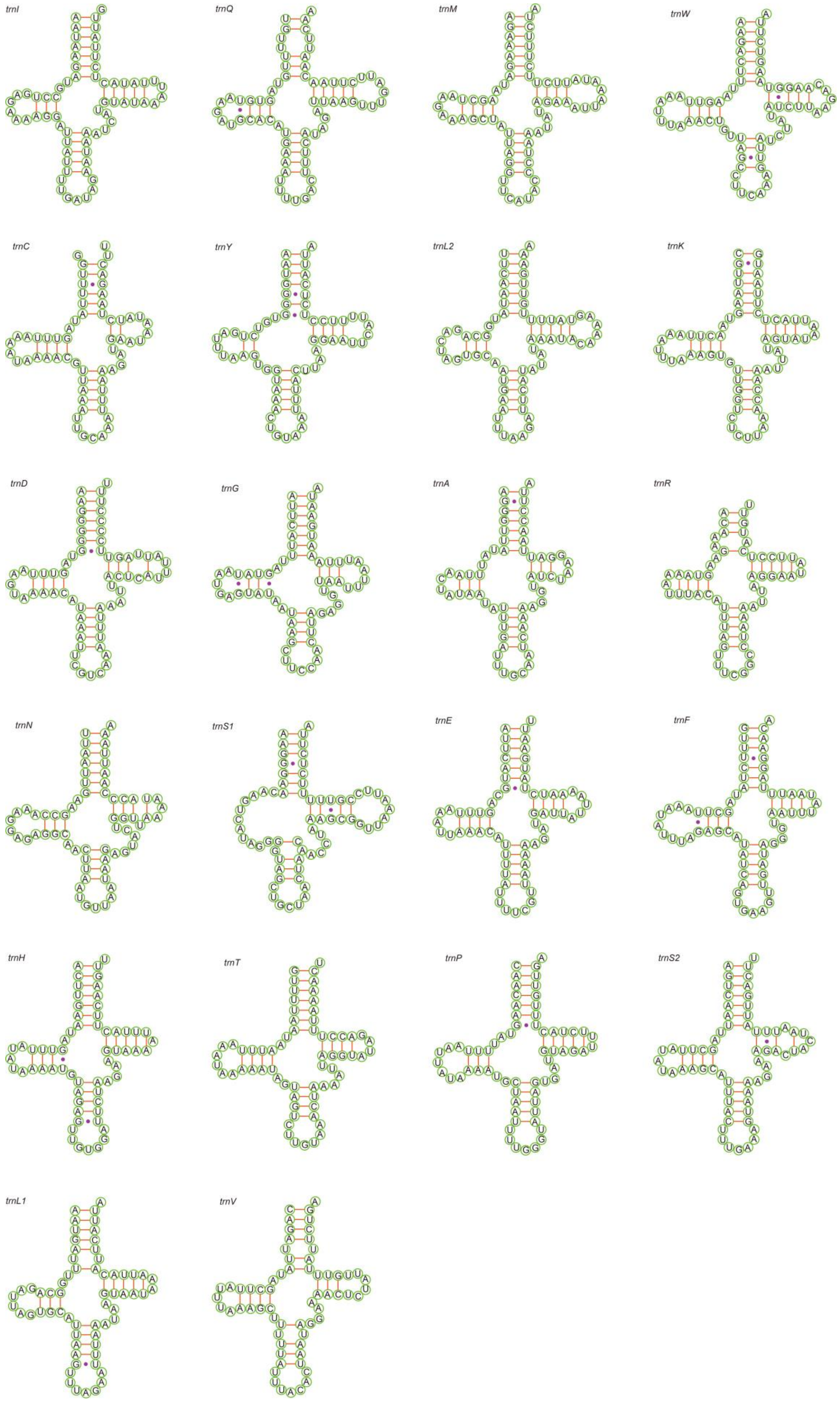

Figure S5. Predicted secondary structures of tRNA genes of *Exitianus nanus*.

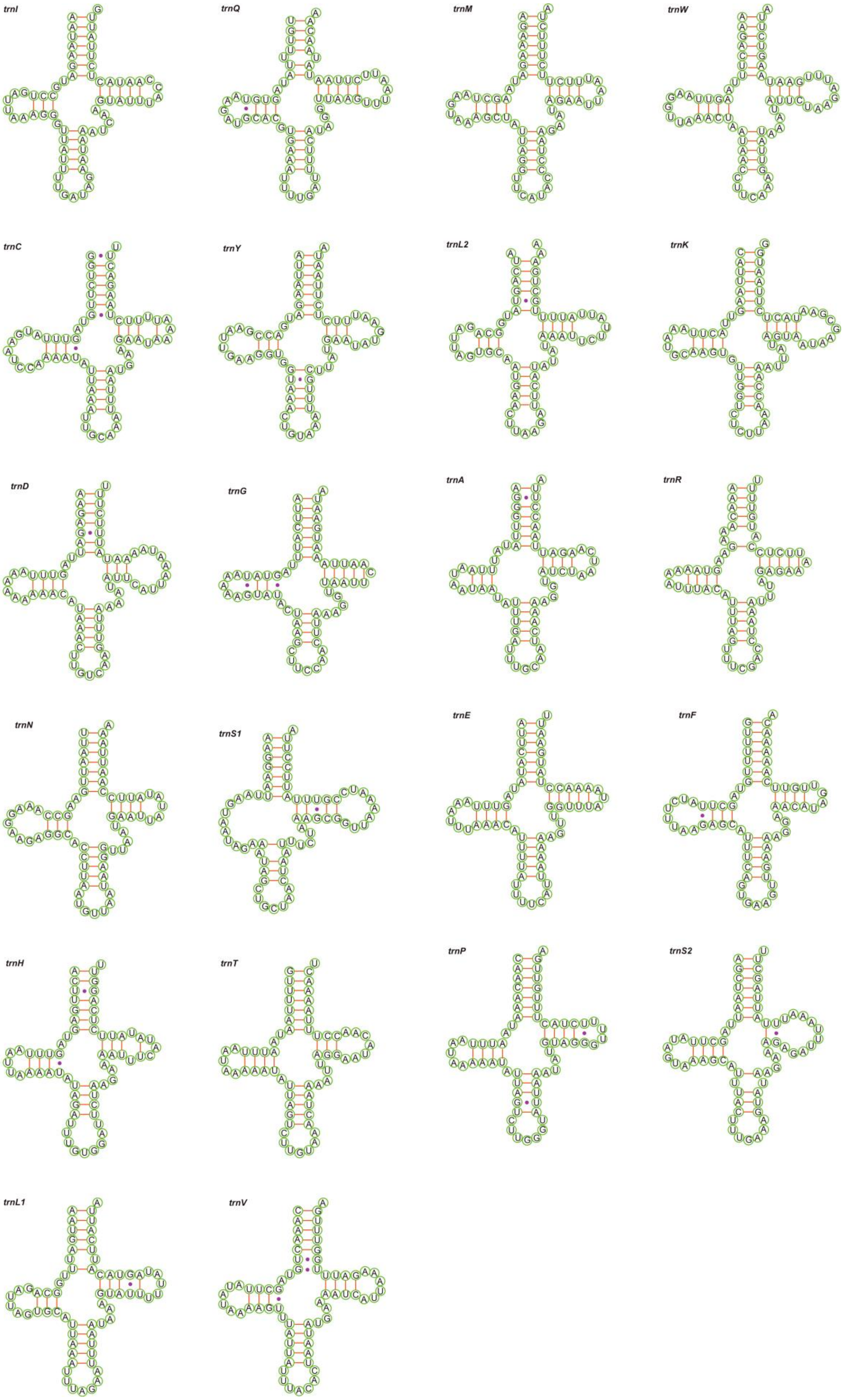

Figure S6. Predicted secondary structures of tRNA genes of *Doratura stylata*.

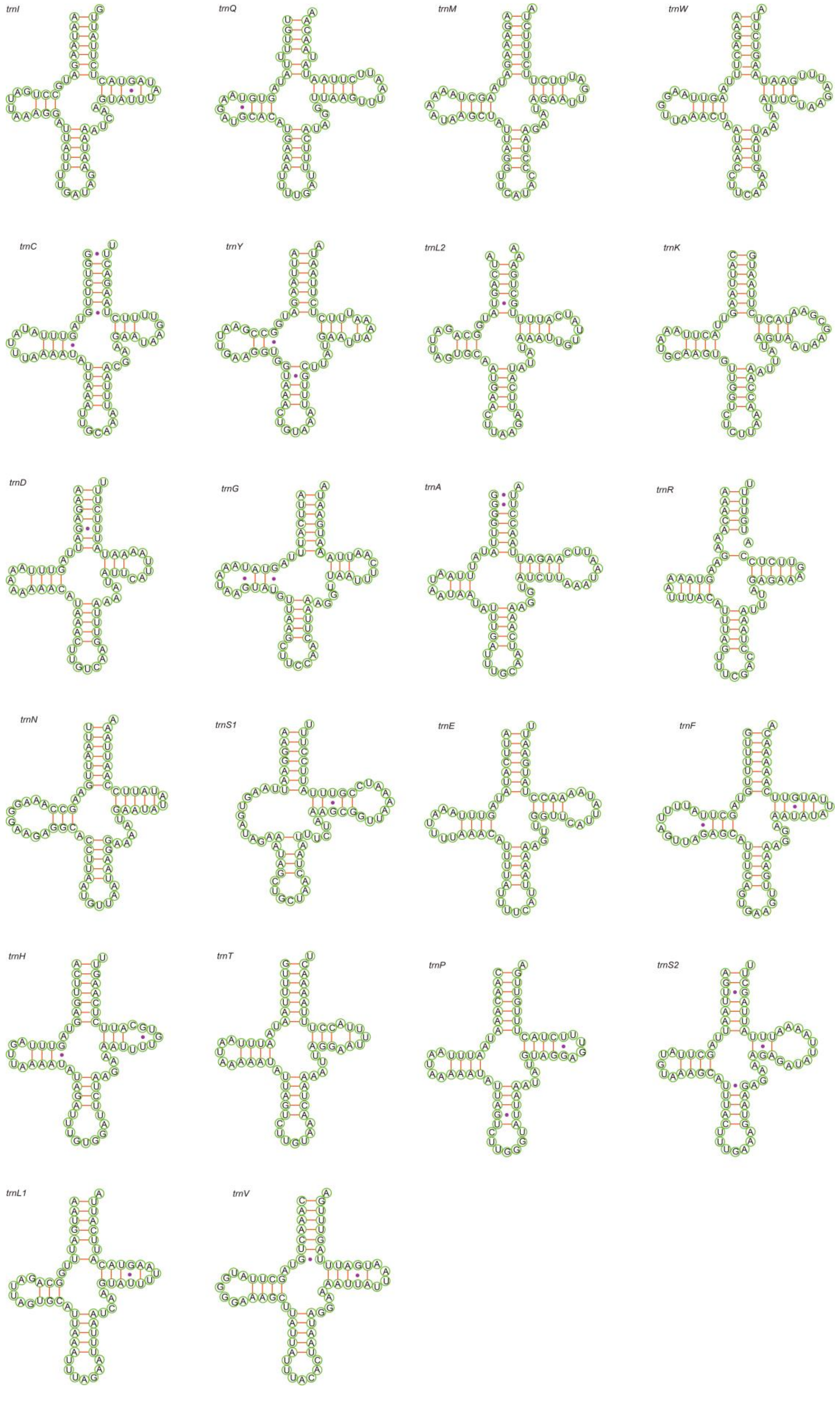

Figure S7. Predicted secondary structures of tRNA genes of *Doratura homophyla*.

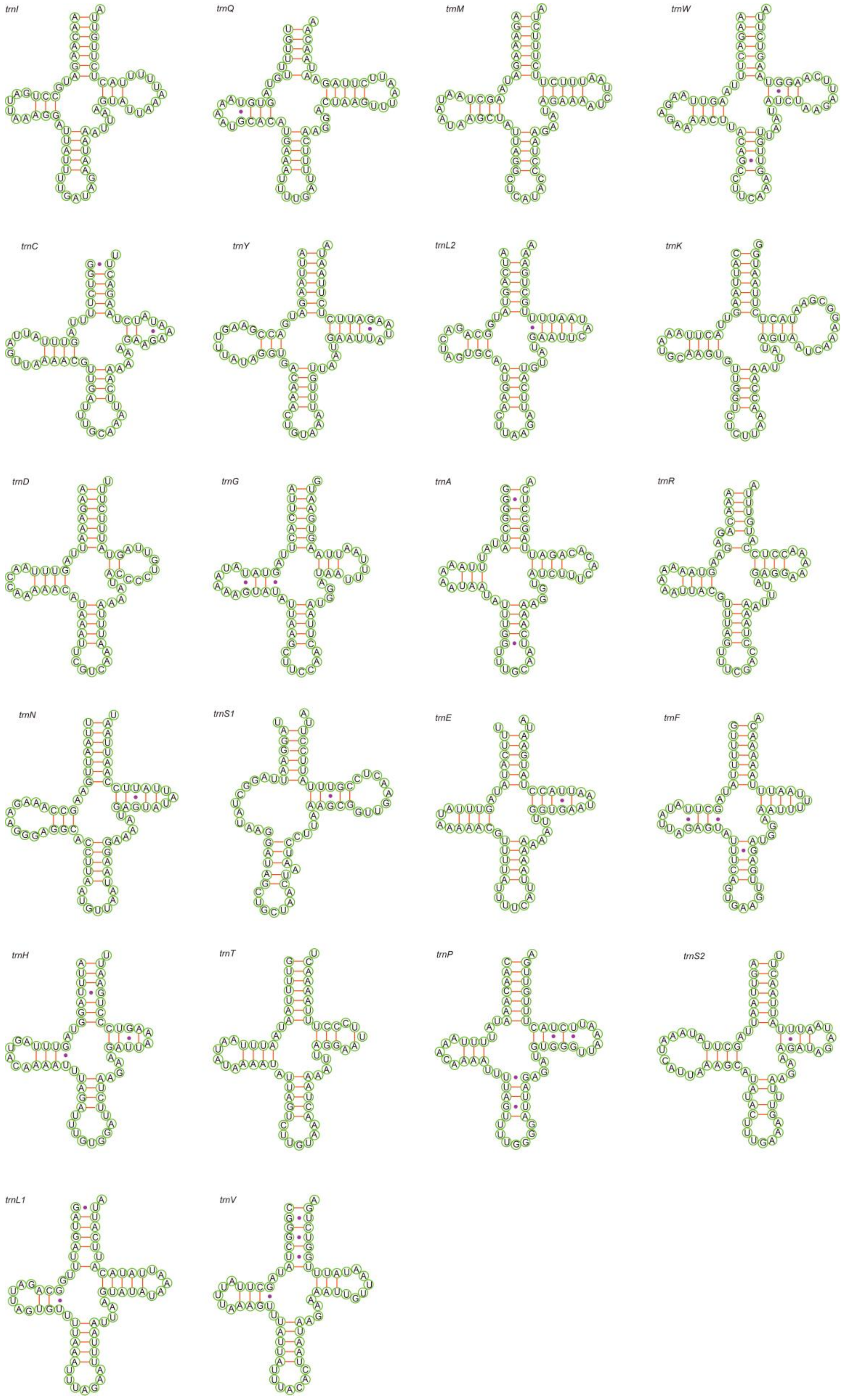

Figure S8. Predicted secondary structures of tRNA genes of *Gurawa minorcephala*.

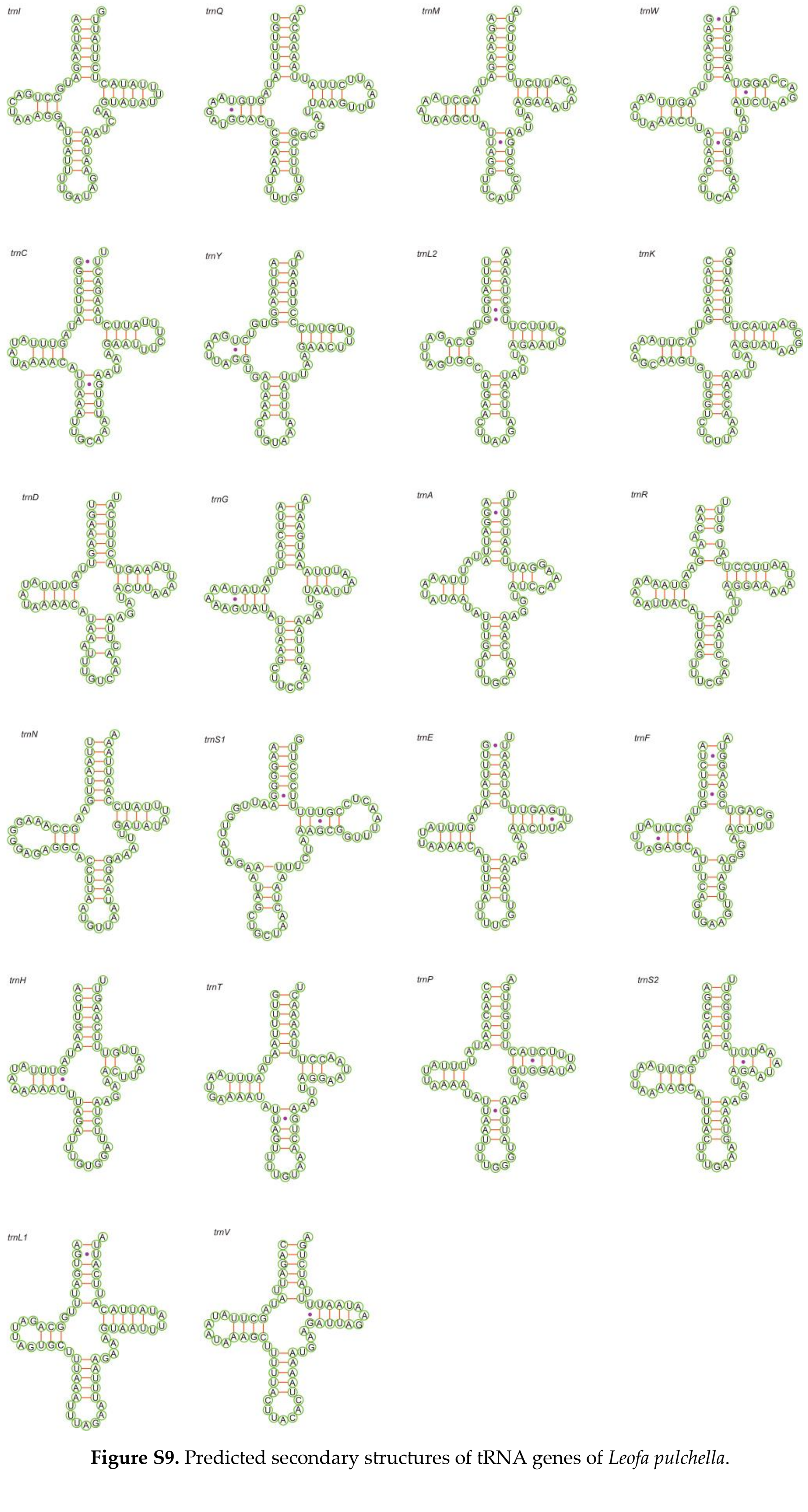

Figure S9. Predicted secondary structures of tRNA genes of *Leofa pulchella*.

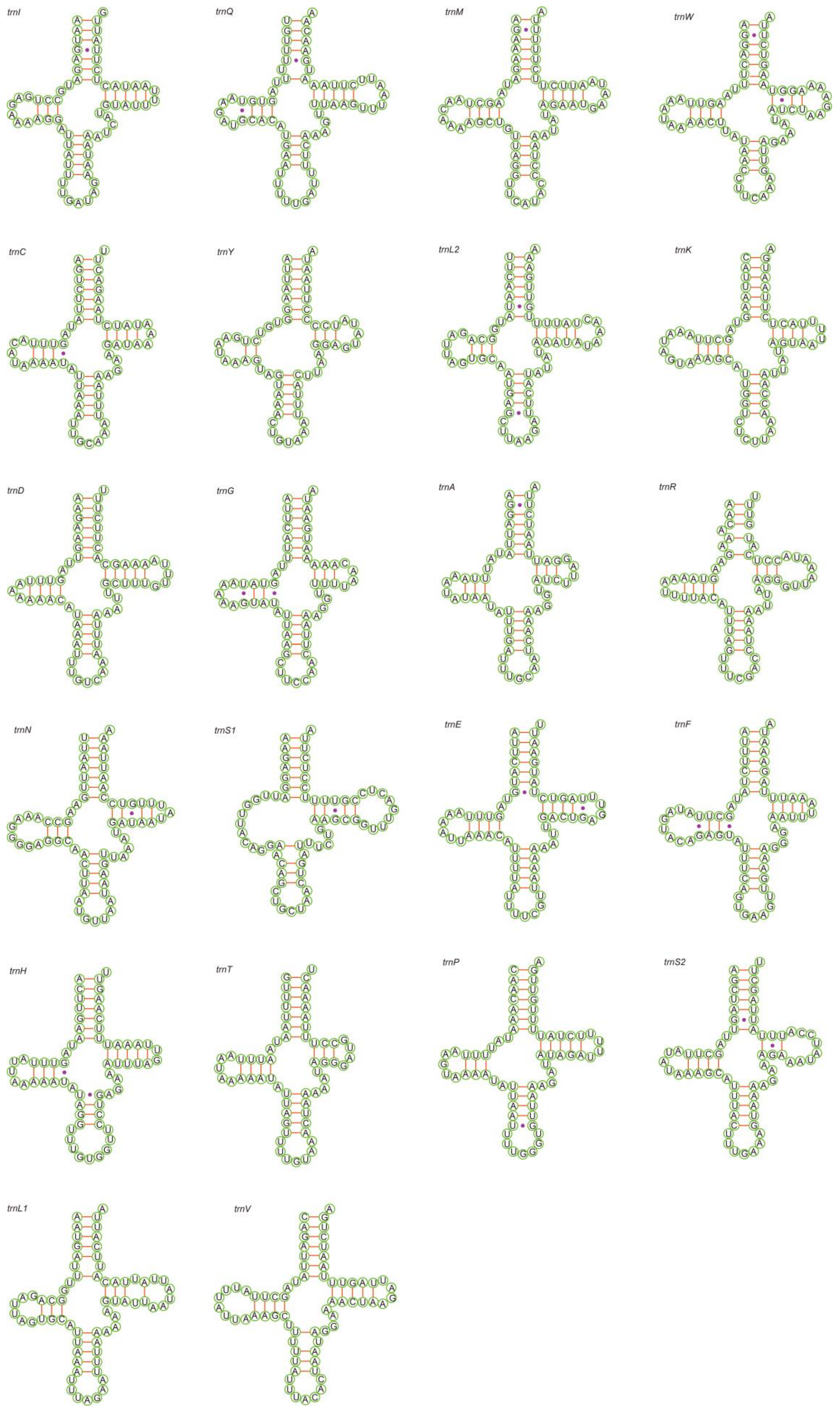

Figure S10. Predicted secondary structures of tRNA genes of *Nephotettix malayanus*.

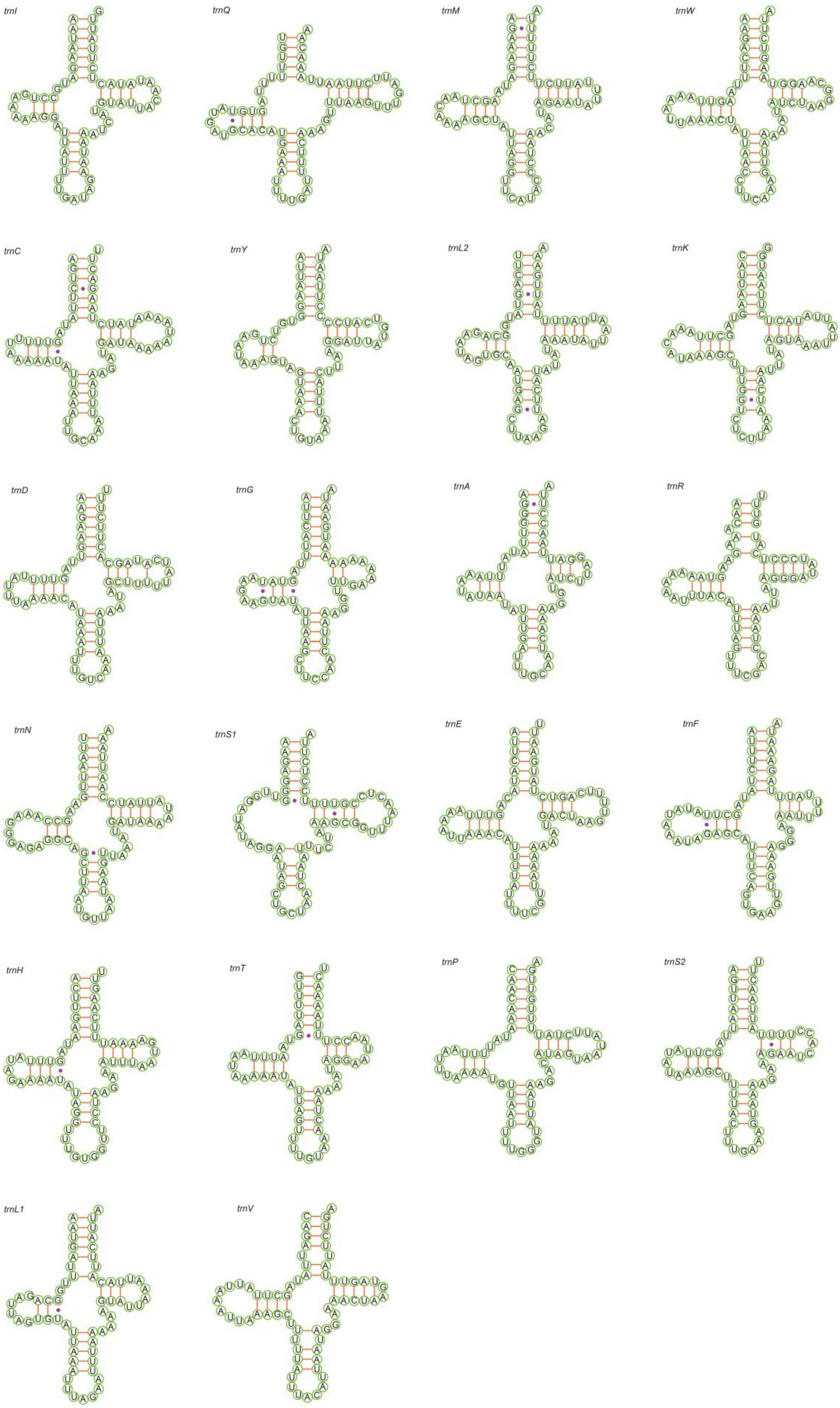

Figure S11. Predicted secondary structures of tRNA genes of *Nephotettix nigropictus*.

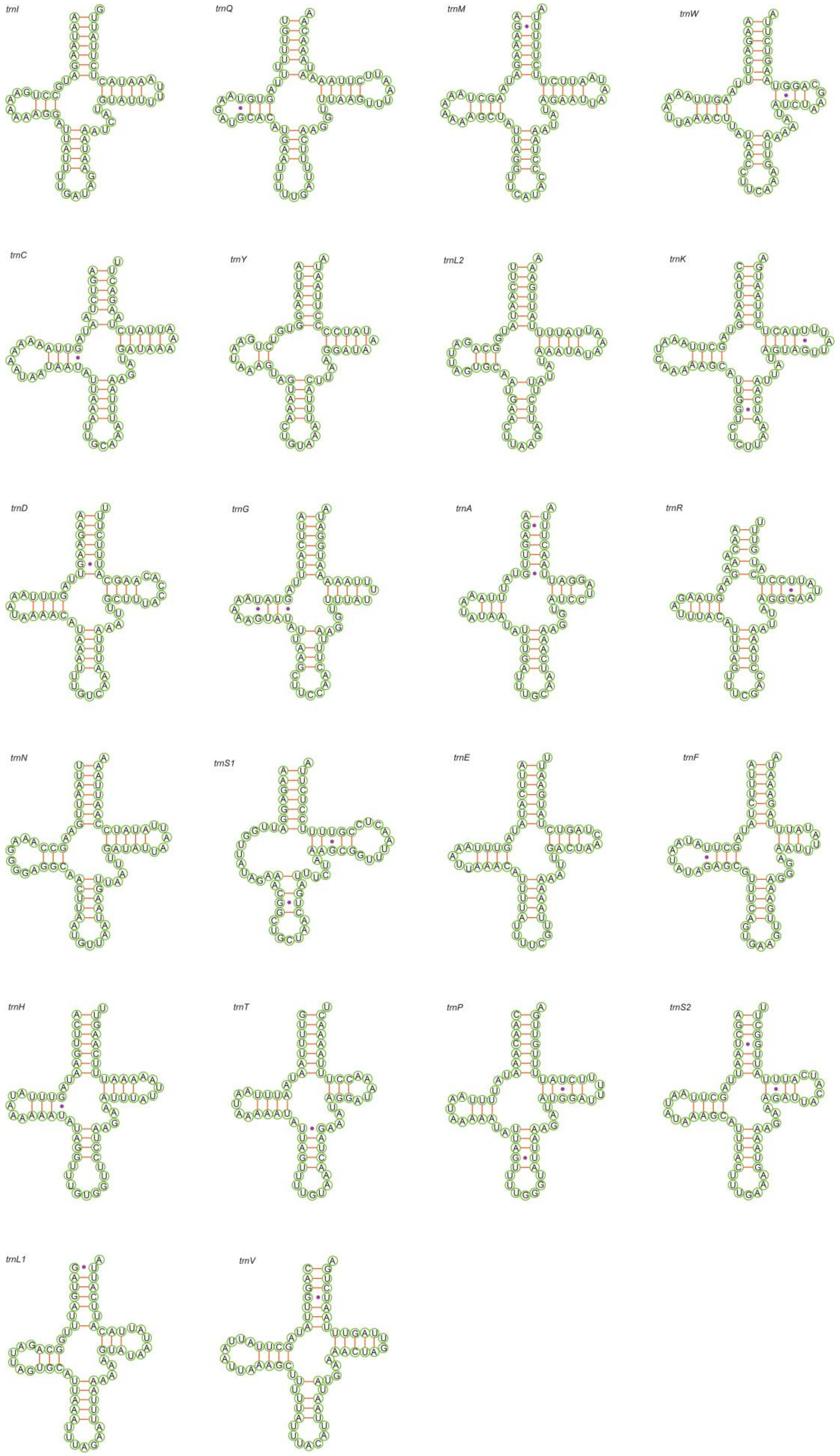

Figure S12. Predicted secondary structures of tRNA genes of *Nephotettix parvus*.

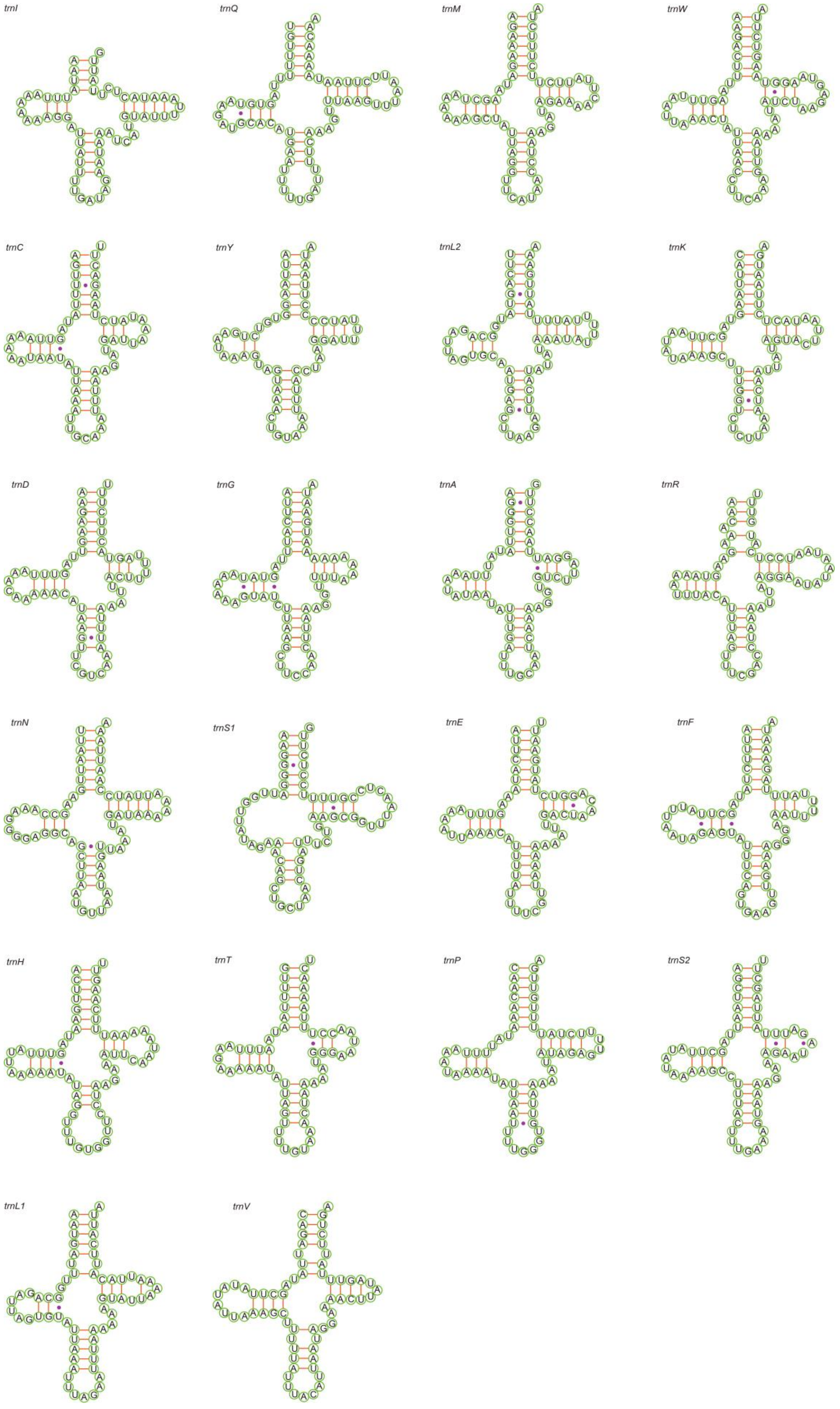

Figure S13. Predicted secondary structures of tRNA genes of *Nephotettix virescens*.

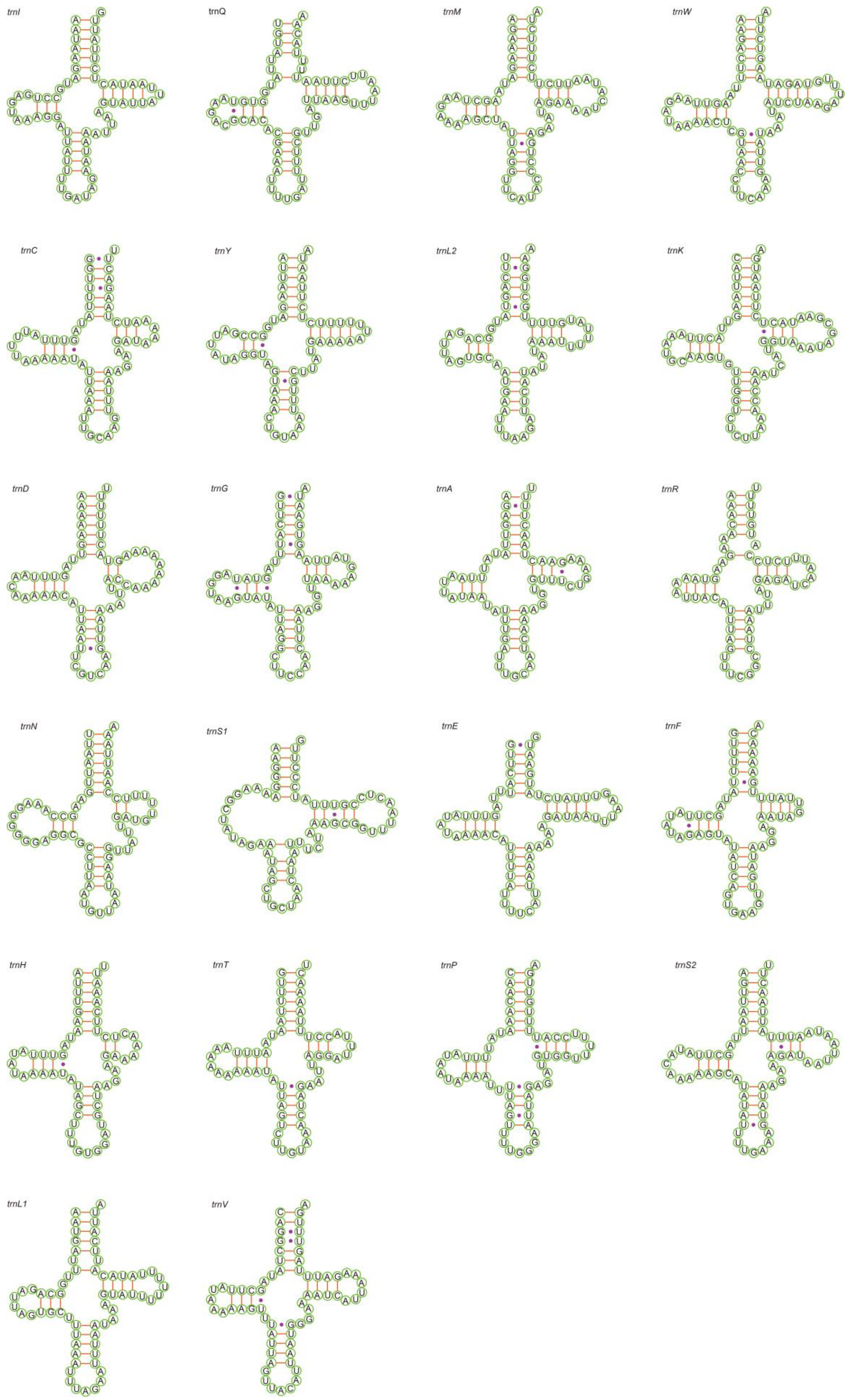

Figure S14. Predicted secondary structures of tRNA genes of *Zahniserius cylindricus*.

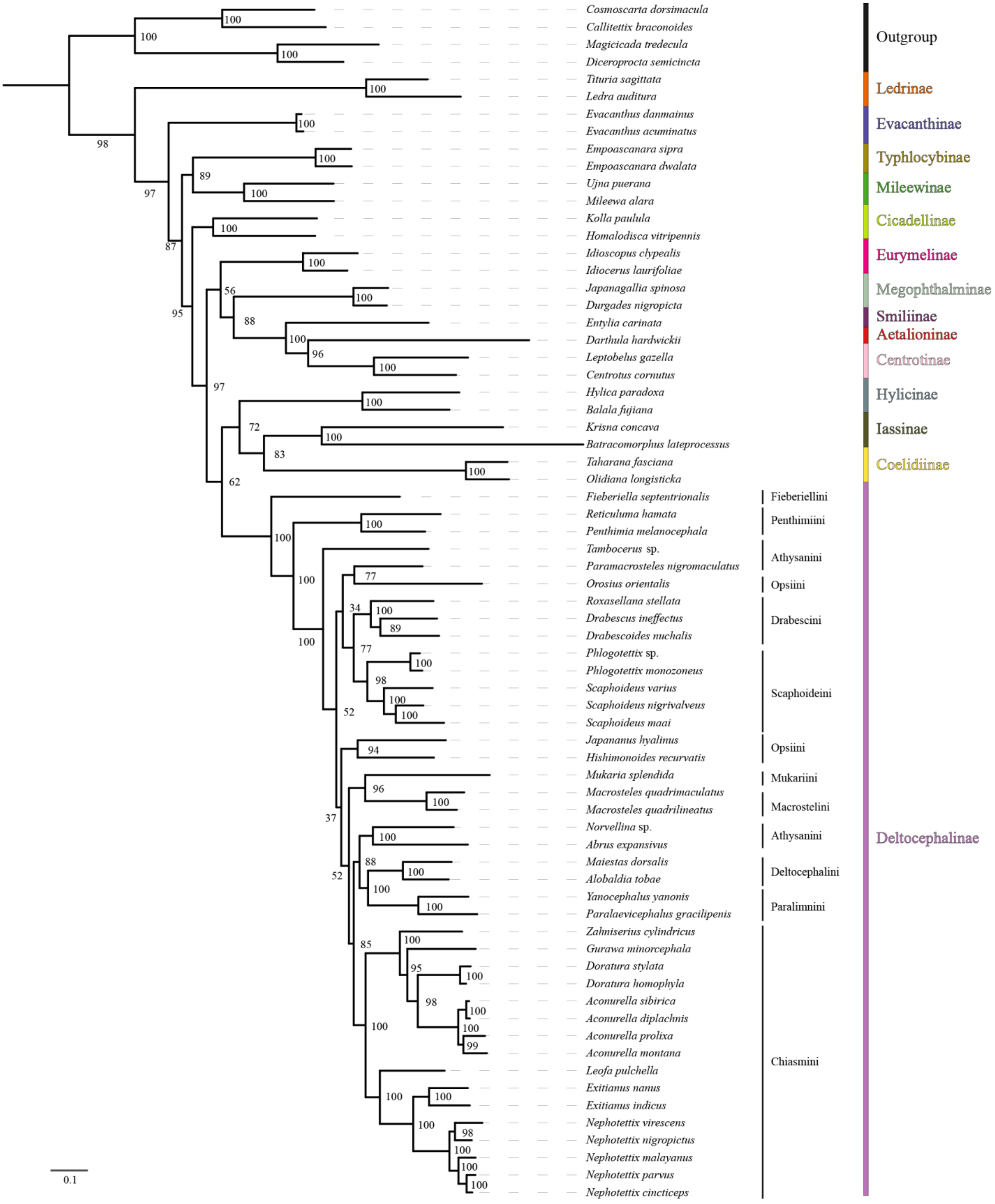

Figure S15. The phylogenetic relationships using the maximum likelihood (ML) analysis method based on the AA datasets. Numbers on each node correspond to the bootstrap values.

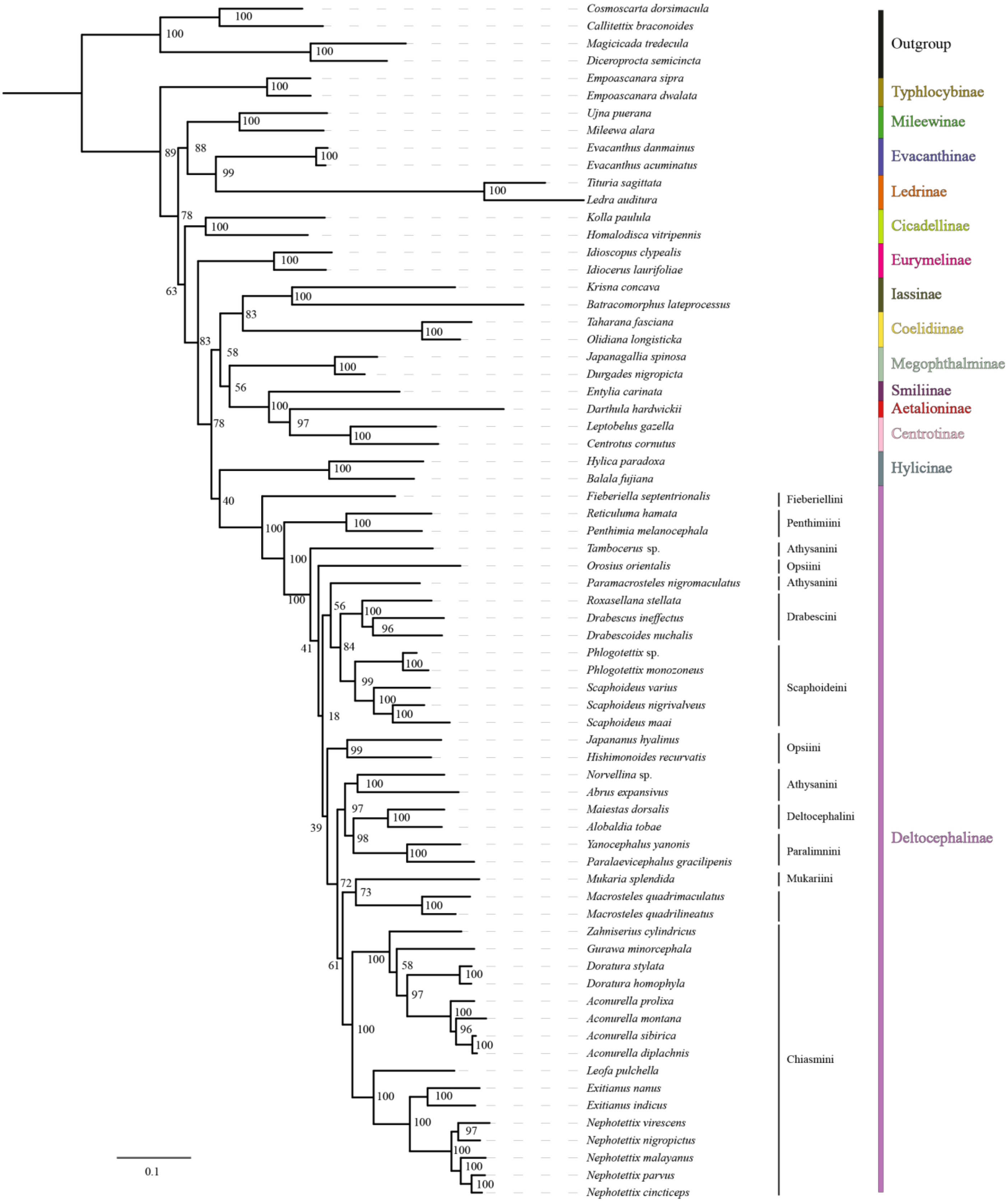

Figure S16. The phylogenetic relationships using the maximum likelihood (ML) analysis method based on the PCG12 datasets. Numbers on each node correspond to the bootstrap values.

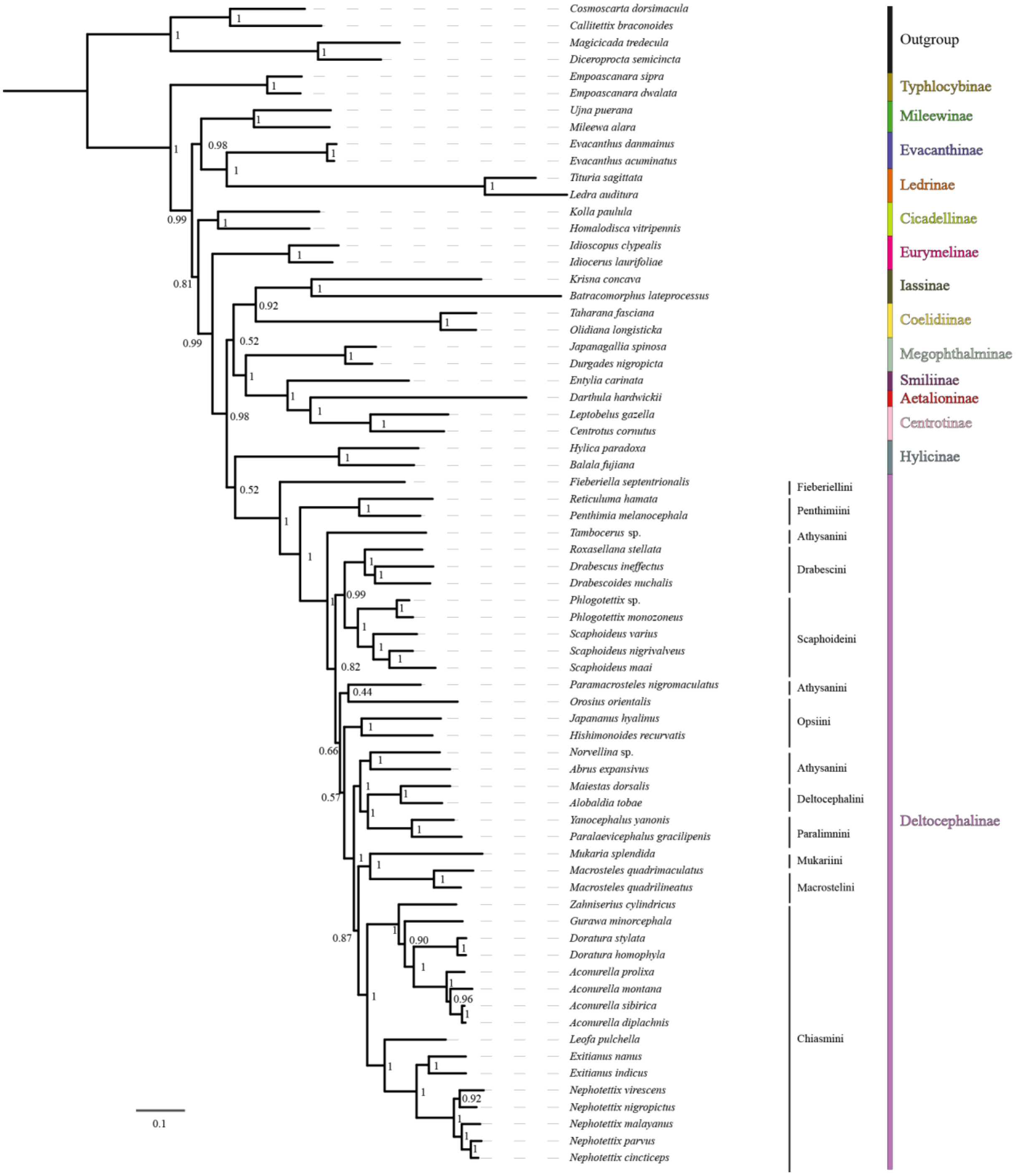

Figure S17. The phylogenetic relationships using the Bayesian inference (BI) analysis method based on the PCG12 datasets. Numbers on each node correspond to the posterior probability (PP) values.

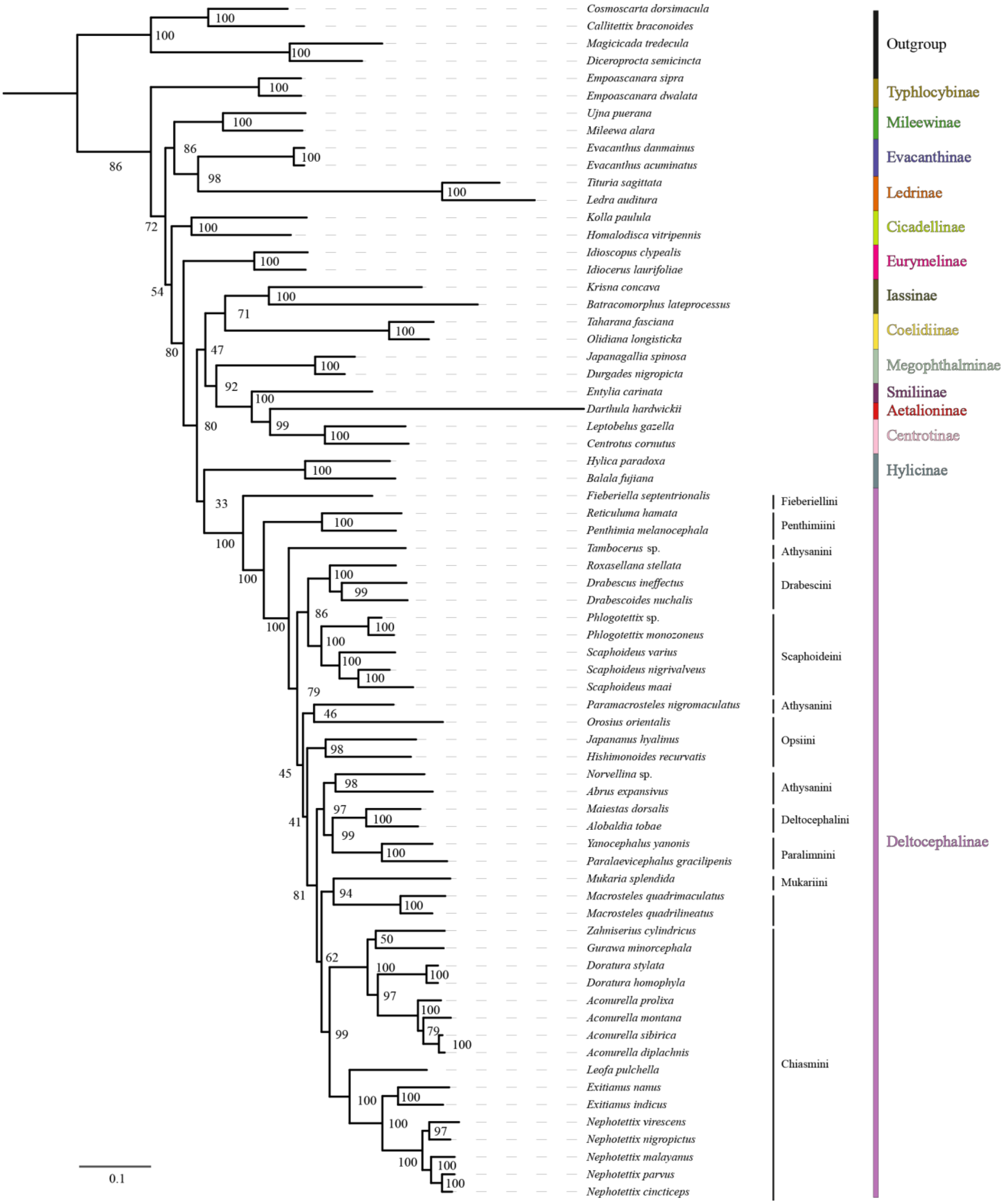

Figure S18. The phylogenetic relationships using the maximum likelihood (ML) analysis method based on the PCG12R datasets. Numbers on each node correspond to the bootstrap values.

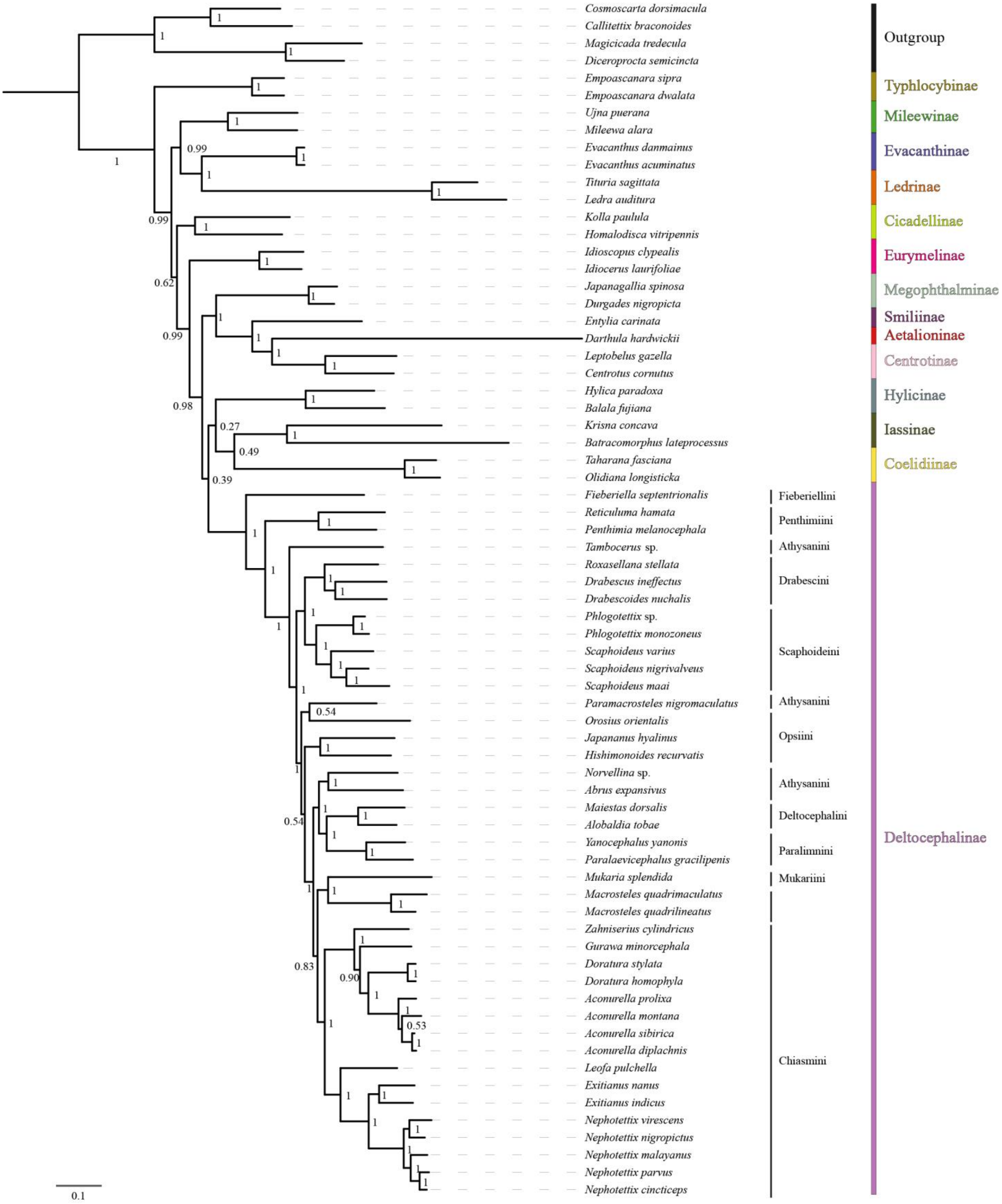

Figure S19. The phylogenetic relationships using the Bayesian inference (BI) analysis method based on the PCG12R datasets. Numbers on each node correspond to the posterior probability (PP) values.

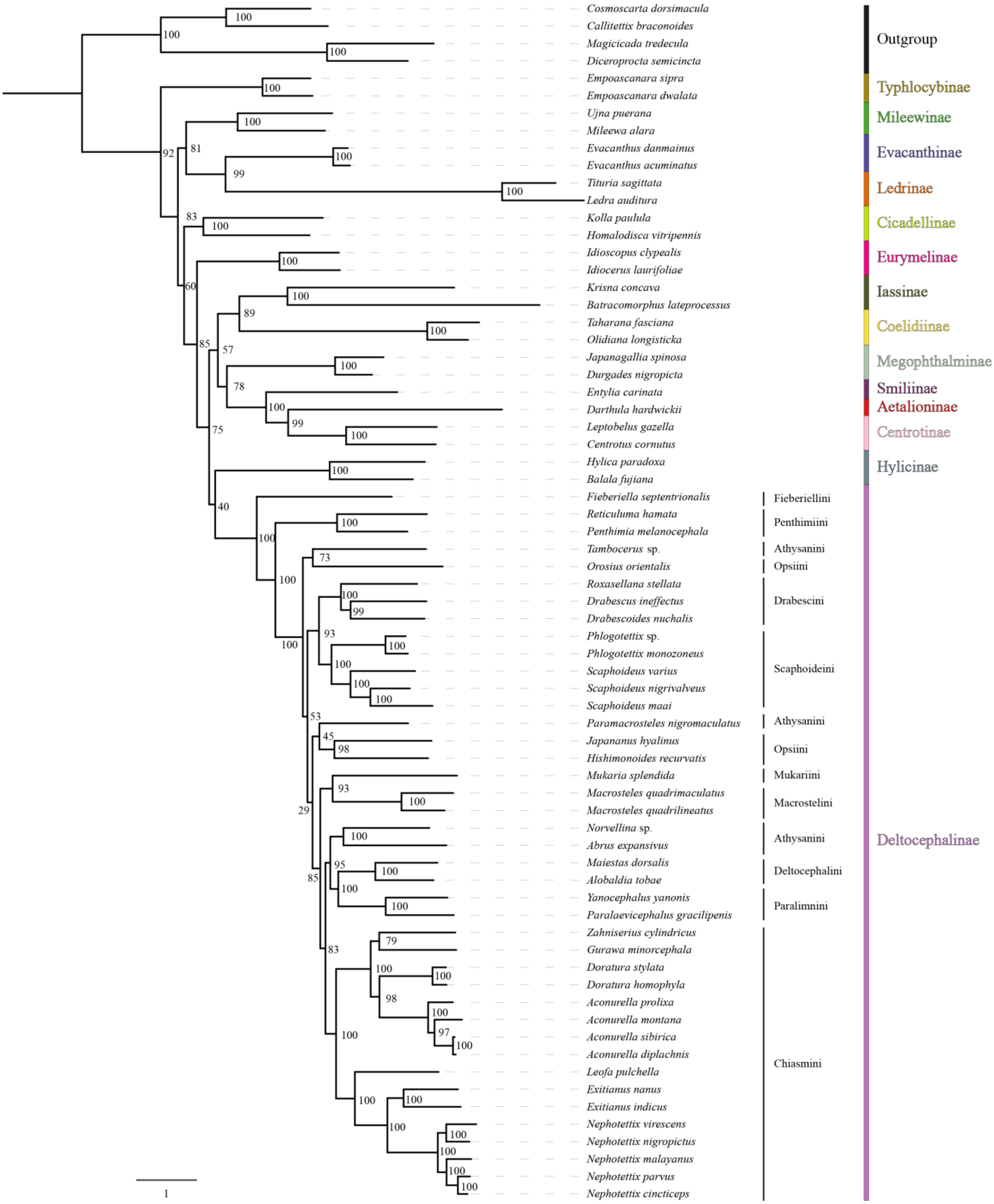

Figure S20. The phylogenetic relationships using the maximum likelihood (ML) analysis method based on the PCG datasets. Numbers on each node correspond to the bootstrap values.

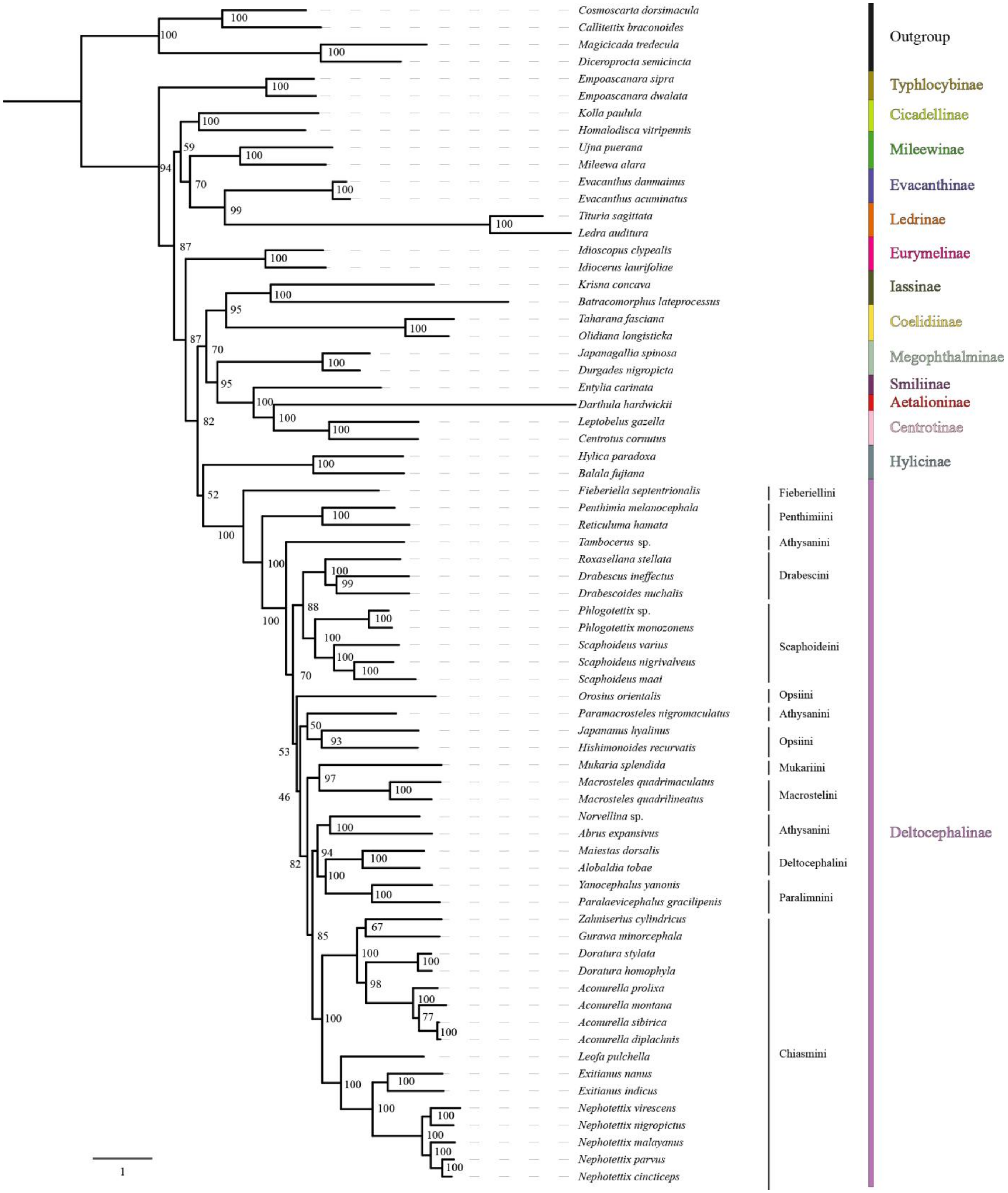

Figure S21. The phylogenetic relationships using the maximum likelihood (ML) analysis method based on the PCGR datasets. Numbers on each node correspond to the bootstrap values.

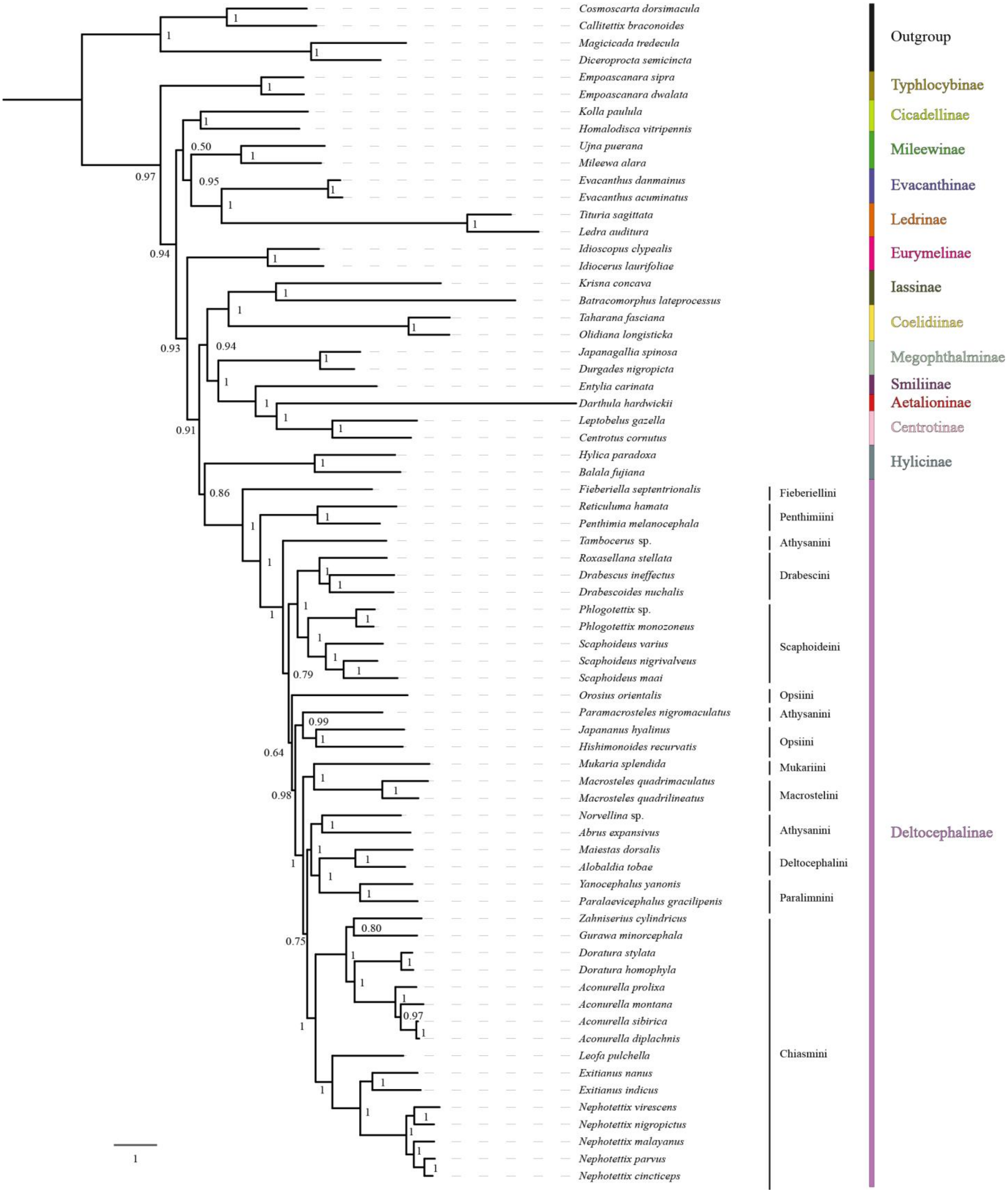

Figure S22. The phylogenetic relationships using the Bayesian inference (BI) analysis method based on the PCGR datasets. Numbers on each node correspond to the posterior probability (PP) values.
