# Supplementary material for: Mitogenomic Analysis and Phylogenetic Implications for the Deltocephaline Tribe Chiasmini (Hemiptera: Cicadellidae: Deltocephalinae)
Source: Insects. 2024 Apr 8;15(4):253. doi: 10.3390/insects15040253 (PMC11050438; doi:10.3390/insects15040253)
Supplement: Supplementary file 1 [file insects-15-00253-s001.zip › insects-2878867-supplementary table.pdf]

Table S1: Best partitioning scheme and models for different datasets selected by PartitionFinder.

Data matrix	Subset	Partitions	Model
**AA–ML**	P1	*ATP6*, *ATP8*, *COX2*, *COX3*, *CYTB*, *ND2*, *ND3*, *ND6*	MTART+I+G+F
	P2	*COXI*	MTART+I+G
	P3	*ND1*, *ND4*, *ND4L*, *ND5*	MTART+I+G+F
**AA–BI**	P1	*ATP6*, *ATP8*, *COX2*, *COX3*, *CYTB*	MTREV+I+G
	P2	*COXI*	MTREV+I+G
	P3	*ND1*, *ND3*, *ND4*, *ND5*	MTREV+I+G
	P4	*ND2*	MTREV+I+G
	P5	*ND4L*, *ND6*	MTREV+I+G
**PCG12–ML**	P1	*ATP6*, *ATP8*, *COXI*, *COX2*, *COX3*, *CYTB*, *ND1*, *ND2*, *ND3*, *ND4*, *ND4L*, *ND5*, *ND6*	GTR+I+G
**PCG12–BI**	P1	*ATP6*, *ATP8*, *COXI*, *COX2*, *COX3*, *CYTB*, *ND1*, *ND2*, *ND3*, *ND4*, *ND4L*, *ND5*, *ND6*	GTR+I+G
**PCG12R–ML**	P1	*ATP6*, *ATP8*, *COXI*, *COX2*, *COX3*, *CYTB*, *ND1*, *ND2*, *ND3*, *ND4*, *ND4L*, *ND5*, *ND6*	GTR+I+G
	P2	*rrnL*, *rrnS*	TVM+I+G
**PCG12R–BI**	P1	*ATP6*, *ATP8*, *COXI*, *COX2*, *COX3*, *CYTB*, *ND1*, *ND2*, *ND3*, *ND4*, *ND4L*, *ND5*, *ND6*	GTR+I+G
	P2	*rrnL*, *rrnS*	GTR+I+G
**PCG–ML**	P1	*ATP6*_codon1	GTR+I+G
	P2	*ND2*_codon2, *ATP8*_codon2, *ND6*_codon2, *ND3*_codon2, *ATP6*_codon2	TVM+I+G
	P3	*COX2*_codon3, *ATP6*_codon3, *COX3*_codon3, *ND3*_codon3	TIM+I+G
	P4	*ATP8*_codon1, *ND3*_codon1, *ND6*_codon1, *ND2*_codon1	GTR+I+G
	P5	*ATP8*_codon3, *CYTB*_codon3, *ND6*_codon3	GTR+G
	P6	*COX1*_codon1	GTR+I+G
	P7	*COX1*_codon2, *COX3*_codon2, *COX2*_codon2, *CYTB*_codon2	TVM+I+G
	P8	*COX1*_codon3	TRN+I+G
	P9	*CYTB*_codon1, *COX3*_codon1, *COX2*_codon1	GTR+I+G
	P10	*ND1*_codon1, *ND4*_codon1, *ND5*_codon1, *ND4L*_codon1	GTR+I+G
	P11	*ND1*_codon2, *ND5*_codon2, *ND4*_codon2, *ND4L*_codon2	GTR+I+G
	P12	*ND1*_codon3	K81UF+I
	P13	*ND2*_codon3	TRN+G
	P14	*ND4L*_codon3, *ND5*_codon3, *ND4*_codon3	TRN+I+G
**PCG–BI**	P1	*ATP6*_codon1	GTR+I+G
	P2	*ND3*_codon2, *ATP6*_codon2, *ATP8*_codon2, *ND6*_codon2, *ND2*_codon2	GTR+I+G
	P3	*COX2*_codon3, *ATP6*_codon3, *ND3*_codon3, *COX3*_codon3	GTR+I+G
	P4	*ATP8*_codon1, *ND6*_codon1, *ND2*_codon1, *ND3*_codon1	GTR+I+G
	P5	*ATP8*_codon3, *ND6*_codon3, *CYTB*_codon3	GTR+G
	P6	*COX1*_codon1	GTR+I+G
	P7	*COX1*_codon2, *COX3*_codon2, *COX2*_codon2, *CYTB*_codon2	GTR+I+G
	P8	*COX1*_codon3	HKY+I+G
	P9	*CYTB*_codon1, *COX3*_codon1, *COX2*_codon1	GTR+I+G
	P10	*ND1*_codon1, *ND4*_codon1, *ND4L*_codon1, *ND5*_codon1	GTR+I+G
	P11	*ND1*_codon2, *ND5*_codon2, *ND4*_codon2, *ND4L*_codon2	GTR+I+G
	P12	*ND1*_codon3	HKY+I+G
	P13	*ND2*_codon3	GTR+G
	P14	*ND4*_codon3, *ND4L*_codon3, *ND5*_codon3	HKY+I+G

**PCGR–ML**	P1	*ATP6*_codon1	GTR+I+G
	P2	*ND2*_codon2, *ATP8*_codon2, *ND6*_codon2, *ATP6*_codon2, *ND3*_codon2	TVM+I+G
	P3	*ATP6*_codon3, *COX3*_codon3, *ND3*_codon3, *COX1*_codon3, *COX2*_codon3	TIM+I+G
	P4	*ATP8*_codon1, *ND3*_codon1, *ND2*_codon1, *ND6*_codon1	GTR+I+G
	P5	*ATP8*_codon3, *CYTB*_codon3, *ND6*_codon3	GTR+G
	P6	*COX1*_codon1	GTR+I+G
	P7	*COX1*_codon2, *COX3*_codon2, *COX2*_codon2, *CYTB*_codon2	TVM+I+G
	P8	*CYTB*_codon1, *COX2*_codon1, *COX3*_codon1	GTR+I+G
	P9	*ND1*_codon1, *rrnS*	TVM+I+G
	P10	*ND1*_codon2, *ND4*_codon2, *ND4L*_codon2, *ND5*_codon2	GTR+I+G
	P11	*ND1*_codon3	K81UF+I
	P12	*ND2*_codon3	TRN+G
	P13	*ND4*_codon1, *ND5*_codon1, *ND4L*_codon1	GTR+I+G
	P14	*ND4L*_codon3, *ND4*_codon3, *ND5*_codon3	TRN+I+G
	P15	*rrnL*	GTR+I+G
**PCGR–BI**	P1	*ATP6*_codon1	GTR+I+G
	P2	*ND3*_codon2, *ATP6*_codon2, *ATP8*_codon2, *ND2*_codon2, *ND6*_codon2	GTR+I+G
	P3	*COX2*_codon3, *ATP6*_codon3, *ND3*_codon3, *COX3*_codon3	GTR+I+G
	P4	*ATP8*_codon1, *ND6*_codon1, *ND2*_codon1, *ND3*_codon1	GTR+I+G
	P5	*ATP8*_codon3, *ND6*_codon3, *CYTB*_codon3	GTR+G
	P6	*COX1*_codon1	GTR+I+G
	P7	*COX1*_codon2, *COX3*_codon2, *COX2*_codon2, *CYTB*_codon2	GTR+I+G
	P8	*COX1*_codon3	HKY+I+G
	P9	*CYTB*_codon1, *COX3*_codon1, *COX2*_codon1	GTR+I+G
	P10	*ND1*_codon1, *rrnS*	GTR+I+G
	P11	*ND1*_codon2, *ND4*_codon2, *ND4L*_codon2, *ND5*_codon2	GTR+I+G
	P12	*ND1*_codon3	HKY+I+G
	P13	*ND2*_codon3	GTR+G
	P14	*ND4*_codon1, *ND4L*_codon1, *ND5*_codon1	GTR+I+G
	P15	*ND4L*_codon3, *ND4*_codon3, *ND5*_codon3	HKY+I+G
	P16	*rrnL*	GTR+I+G
